# Mn^3+^-rich oxide/persistent luminescence nanoparticles achieve light-free generation of singlet oxygen and hydroxyl radicals for responsive imaging and tumor treatment

**DOI:** 10.7150/thno.62437

**Published:** 2021-05-25

**Authors:** Dandan Ding, Yushuo Feng, Ruixue Qin, Shi Li, Lei Chen, Jinpeng Jing, Chutong Zhang, Wenjing Sun, Yimin Li, Xiaoyuan Chen, Hongmin Chen

**Affiliations:** 1 State Key Laboratory of Molecular Vaccinology and Molecular Diagnostics & Center for Molecular Imaging and Translational Medicine, School of Public Health, Xiamen University, Xiamen 361102, China.; 2 Department of Radiation Oncology, Cancer Center, the First Affiliated Hospital of Xiamen University, Xiamen 361003, China.; 3 Departments of Diagnostic Radiology and Surgery, Clinical Imaging Research Centre, Centre for Translational Medicine, Nanomedicine Translational Research Program, NUS Center for Nanomedicine, Yong Loo Lin School of Medicine, Departments of Chemical and Biomolecular Engineering, and Biomedical Engineering, Faculty of Engineering, National University of Singapore, Singapore.

**Keywords:** Mn^3+^-rich oxide, X-ray excited persistent luminescence, tumor environment, chemodynamic therapy, responsive imaging

## Abstract

X-ray excited persistent luminescence (XEPL) imaging has attracted increasing attention in biomedical imaging due to elimination of autofluorescence, high signal-to-noise ratio and repeatable activation with high penetration. However, optical imaging still suffers from limited for high spatial resolution.

**Methods:** Herein, we report Mn^3+^-rich manganese oxide (MnO_x_)-coated chromium-doped zinc gallogermanate (ZGGO) nanoparticles (Mn-ZGGOs). Enhanced XEPL and magnetic resonance (MR) imaging were investigated by the decomposition of MnO_x_ shell in the environment of tumors. We also evaluated the tumor cell-killing mechanism by detection of reactive oxygen (ROS), lipid peroxidation and mitochondrial membrane potential changes *in vitro*. Furthermore, the *in vivo* biodistribution, imaging and therapy were studied by U87MG tumor-bearing mice.

**Results:** In the tumor region, the MnO_x_ shell is quickly decomposed to produce Mn^3+^ and oxygen (O_2_) to directly generate singlet oxygen (^1^O_2_). The resulting Mn^2+^ transforms endogenous H_2_O_2_ into highly toxic hydroxyl radical (·OH) via a Fenton-like reaction. The Mn^2+^ ions and ZGGOs also exhibit excellent T_1_-weighted magnetic resonance (MR) imaging and ultrasensitive XEPL imaging in tumors.

**Conclusion:** Both the responsive dual-mode imaging and simultaneous self-supplied O_2_ for the production of ^1^O_2_ and oxygen-independent ·OH in tumors allow for more accurate diagnosis of deep tumors and more efficient inhibition of tumor growth without external activation energy.

## Introduction

Versus traditional optical technologies, persistent luminescence efficiently avoids the tissue autofluorescence interference due to lack of external illumination from *in situ* excitation 1, 2. In particular, near-infrared (NIR) emitting persistent luminescence is a promising candidate for *in vivo* imaging due to deeper tissue penetration depth and higher signal-to-noise ratio 3, 4. The excitation source is mainly UV light 5, which cannot repeatedly stimulate the persistent luminescence to realize *in vivo* renewable imaging due to a poor tissue penetration depth 6. Recently, white and red light were used as an excitation source to reactivate persistent luminescence for *in vivo* imaging 7-9, but the light penetration is still limited for further bio-applications. X-rays have been widely used in deep imaging and therapy in clinic, including as excitation sources for *in vivo* recharged deep tissue imaging 10-14.

Researches had demostrated that ZGGO persistent luminescence nanocomposite could load therapeutic drug for realizing long-term drug tracking and significant tumor therapeutic effect 15, 16. Moreover, the persistent luminescence materials could be employed as photodynamic therapeutic agents. Rencent research showed the ZnGa_1.996_O_4_:Cr_0.004_ persistent luminescence nanomaterial was an effective excitation source to achieve repeatable photodynamic therapy *in vivo* and effectively inhibited tumor growth 17. Our previous study also demostrated the Zn_3_Ga_2_GeO_8_:Cr^3+^,Yb^3+^,Er^3+^@mSiO_2_ persistent luminescence nanomaterials realized X-ray induced ultrasensitive persistent luminescence imaging and effective inhibition of orthotopic hepatic tumors 14.

However, persistent luminescence suffers from poor spatial resolution. In contrast, magnetic resonance (MR) imaging offers detailed three-dimensional anatomical images 18. Thus, multi-functional and/or multi-modal imaging probes can combine these strengths. Manganese oxides have been employed as an excellent choice for cancer diagnostics 19-25. Manganese oxides (MnO_x_) can react with the environment in tumor regions, i.e. low pH, overexpressed glutathione (GSH), and high hydrogen peroxide (H_2_O_2_) level to release Mn^2+^ and O_2_ 26-32. Our previous study demonstrated that magnetic manganese oxide sweetgum-balls could be used as drug carriers to realize enhanced tumor theranostics 33. Recent studies showed that MnO_x_ could degrade to produce Mn^2+^ and react with H_2_O_2_ by generating limited hydroxyl radical (·OH) for chemodynamic therapy (CDT) 34. CDT is a novel anticancer strategy due to generation of highly toxic reactive oxygen 35, 36. The Mn^3+^-contained in MnO_x_ has strong catalytic ability to achieve the light-free generation of ^1^O_2_ to boost dynamic therapy efficacy 37. Thus, this Mn approach is a promising strategy to develop nanotheranostics that can generate several kinds of ROS synchronously from multiple reactants for good therapeutic effects.

**Scheme 1:**
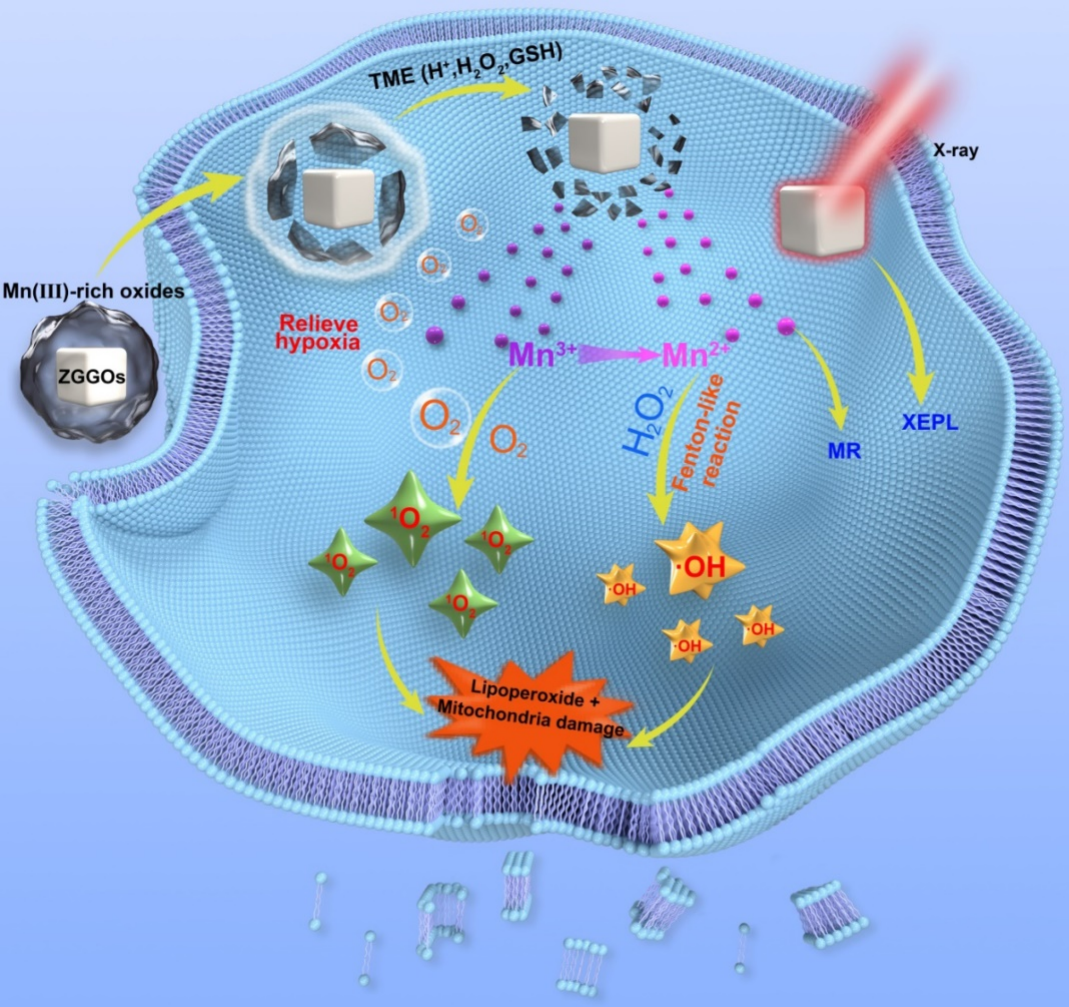
Schematic diagrams of Mn^3+^-rich oxide/persistent luminescence nanoparticles for light-free generation of singlet oxygen and hydroxyl radicals for responsive imaging and tumor treatment.

Herein, we present a facile method to prepare Mn^3+^-rich MnO_x_ coatings on ZGGO nanoparticles (Mn-ZGGOs) (Scheme 1). The environment in tumor regions stimulated the decomposition of MnO_x_ coating to release ions and persistent luminescence nanoparticles. Investigations *in vitro* and *in vivo* showed that the MnO_x_ shell could simultaneously release Mn^3+^ to accelerate endogenous O_2_ into highly toxic ^1^O_2_ and generate more Mn^2+^. The generated Mn^2+^ then transforms endogenous H_2_O_2_ into the highly toxic ·OH. Thus, effective tumor inhibition *in vivo* was attributed to the concurrent generation of two different ROS to achieve parallel CDT. At the same time, the released Mn^2+^ could be used as MR imaging agent with higher spatial resolution. And exposed ZGGOs was also an ultrasensitive XEPL imaging agent with higher tissue penetration depth in tumors.

## Methods

### Synthesis of Mn-ZGGOs

10 mg ZGGO nanoparticles were dispersed in 10 mL ethanol by sonication for 1 h, and then centrifuged and washed with deionized water. Next, KMnO_4_ (20 mg) aqueous solution was dropwise added into the suspension of ZGGO under sonication. After 2 h, the precipitate was obtained by centrifugation at 10000 rpm.

### *In vitro* X-ray recharged persistent luminescence imaging

Mn-ZGGOs solution ([Mn] = 0.2 mg/mL, 200 μL) was excited by X-ray (0.1 Gy). After 60 s, the GSH solution (10 mM, 20 μL) was added into Mn-ZGGOs, and XEPL signals with GSH or without GSH were collected on an IVIS Lumina II imaging system in the BLI mode at different time points, respectively. After 2 h, Mn-ZGGOs solution was re-irradiated by X-rays and the recharged images were acquired.

### Measurement of ·OH

25 mM NaHCO_3_ solution containing Mn-ZGGOs NPs and different concentrations of GSH (0, 0.5, 1.0 and 10 mM) was mixed for 30 min. After centrifugation, 10 μg/mL MB and 10 mM H_2_O_2_ were added to the supernatant. Then, the mixture was incubated at 37 °C for 30 min, and the absorbance change of MB at 665 nm was measured.

### Measurement of ^1^O_2_

Mn-ZGGOs was dissolved in 0.5 mL of PBS with different pH values (7.4, 6.5, and 5.6) and then 5 μL of SOSG (500 μM, dissolved in methanol) was added. After incubation for 30 min, the mixture was centrifuged to remove the unreacted Mn-ZGGOs to avoid the interference of Mn-ZGGOs on SOSG fluorescence.

### Generation of O_2_

Mn-ZGGOs ([Mn] = 0, 0.5 mM) were added into 1 mL PBS solution (pH=5.6) with H_2_O_2_ of 10 mM. The produced O_2_ was detected by the fluorescence change of [Ru(dpp)_3_]Cl_2_ probe.

### GSH-activated MR imaging performance

The Mn-ZGGOs NPs aqueous solutions with different Mn concentrations were mixed with GSH for 10 min, and then the MRI analysis was performed on 9.4 T clinical MR imaging.

### *In vitro* cytotoxicity

The cytotoxicity of the as-prepared Mn-ZGGOs was investigated using a standard MTT assay. U87MG cells and L02 cells (1 × 10^4^ cells per well) were seeded into 96-well plates and allowed to grow overnight. The cells were incubated with Mn-ZGGOs at different concentrations for 24 h. After that, 10 μL of MTT was added to each well. After incubation for 4 h, the medium was removed, and 150 μL of DMSO was added to dissolve the emerging formazan crystals. The absorbance at 570 nm was measured with a multi-detection microplate reader.

### *In vitro* generation of ROS

U87MG cells (2 × 10^5^) were seeded into glass bottom dishes (35 mm × 10 mm) and allowed to grow overnight. Then cells were replenished with fresh medium containing Mn-ZGGOs ([Mn] = 0, 10 and 20 μg/mL). After incubation for 24 h, the cells were washed with PBS for three times and stained with Hoechst 33342 (10 μg/mL) for 20 min at 37 °C. After washing with PBS for three times, the cells were further stained with SOSG (5 μM) or DCFH-DA (5 μM) for 20 min at 37 °C. Subsequently, those cells were imaged by Olympus FV1200 laser confocal scanning microscope after washing by PBS.

### Intracellular lipoperoxide evaluation

U87MG cells (2 × 10^5^) were seeded into glass bottom dishes (35 mm × 10 mm) and allowed to grow overnight. The fresh medium containing Mn-ZGGOs ([Mn] = 0 and 20 μg/mL) was added. After incubation for 24 h, the cells were washed with PBS for three times and stained with Hoechst 33342 (10 μg/mL) for 20 min at 37 °C. After washing with PBS for three times, the cells were stained with a lipoperoxide indicator, DOPIBY C11 (5 μM) for 20 min at 37 °C. The intracellular lipoperoxide was monitored using Olympus FV1200 laser confocal scanning microscope after washing by PBS.

### Mitochondrial membrane potential damage evaluation

U87MG cells (2 × 10^5^) were seeded into glass bottom dishes (35 mm × 10 mm) and allowed to grow overnight. The fresh medium containing Mn-ZGGOs ([Mn] = 10, 20 μg/mL) was added. After incubation for 24 h, the cells were washed with PBS for three times and stained with Hoechst 33342 (10 μg/mL) for 20 min at 37 °C. After washing with PBS for three times, the cells were stained with JC-1 dye (5 μM) for 20 min at 37 °C. The damage of mitochondrial membrane potential was observed using Olympus FV1200 laser confocal scanning microscope after washing by PBS.

### *In vitro* O_2_ generation

U87MG cells (2 × 10^5^) were seeded into glass bottom dishes (35 mm × 10 mm) and allowed to grow overnight. The fresh medium containing Mn-ZGGOs ([Mn] = 20 μg/mL) was added. After incubation for 0, 4, 8 and 24 h, respectively, the cells were washed with PBS for three times and stained with Hoechst 33342 (10 μg/mL) for 20 min at 37 °C. After washing with PBS for three times, the cells were stained with [Ru(dpp)_3_]Cl_2_ (10 μg/mL) for 20 min at 37 °C. The production of O_2_ was evaluated using Olympus FV1200 laser confocal scanning microscope after washing by PBS.

### *In vivo* circulation and biodistribution

Balb/c mice bearing U87MG tumors were intravenously injected with Mn-ZGGOs ([Mn] = 1 mg/kg). The tumors and main organs including hearts, livers, spleens, lungs and kidneys, and blood were collected at varied time (1, 2, 4, 8, 12, 24 and 48 h) after injection and were weighed and digested using HNO_3_-H_2_O_2_ mixture. The Mn content in all samples was measured by ICP-MS.

### *In vivo* XEPL imaging

After X-ray irradiation for 1 min, Mn-ZGGOs ([Mn] = 1 mg/kg) was intravenously injected into U87MG tumor-bearing mice. XEPL images were collected using an IVIS *in vivo* imaging system and the tumors were activated by X-ray for another 1 min before acquiring XEPL imaging at different times (0, 2, 4, 8, 12 and 24 h) post-injection.

### *In vivo* MR imaging

To confirm the activatable MR imaging effect in tumors, Mn-ZGGOs NPs ([Mn] = 1 mg/kg) were intravenously injected into mice bearing U87MG tumors, and then the MR images were obtained at different time points (0, 2, 4 h, 8, 12 and 24 h) using small animal MR imaging system (9.4T).

### *In vivo* tumor inhibition

When the tumor size reached to ~ 60 mm^3^, the U87MG tumor-bearing mice were randomized into various groups (n = 5) with different treatments: (1) PBS, (2) ZGGO (6.3 mg/kg), (3) Mn-ZGGOs (8.9 mg/kg, [Mn] = 1 mg/kg), (4) 2×Mn-ZGGOs (17.8 mg/kg, [Mn] = 2 mg/kg). The mice were intravenously injected with different formulations every third day for four times.

### Statistical analysis

All data were presented as mean ± standard deviation. Comparison of the data were conducted with a Student's t-test (*: P < 0.05, **: P < 0.01, and ***: P < 0.001).

## Results and Discussion

### Synthesis and characterization

**Figure 1:**
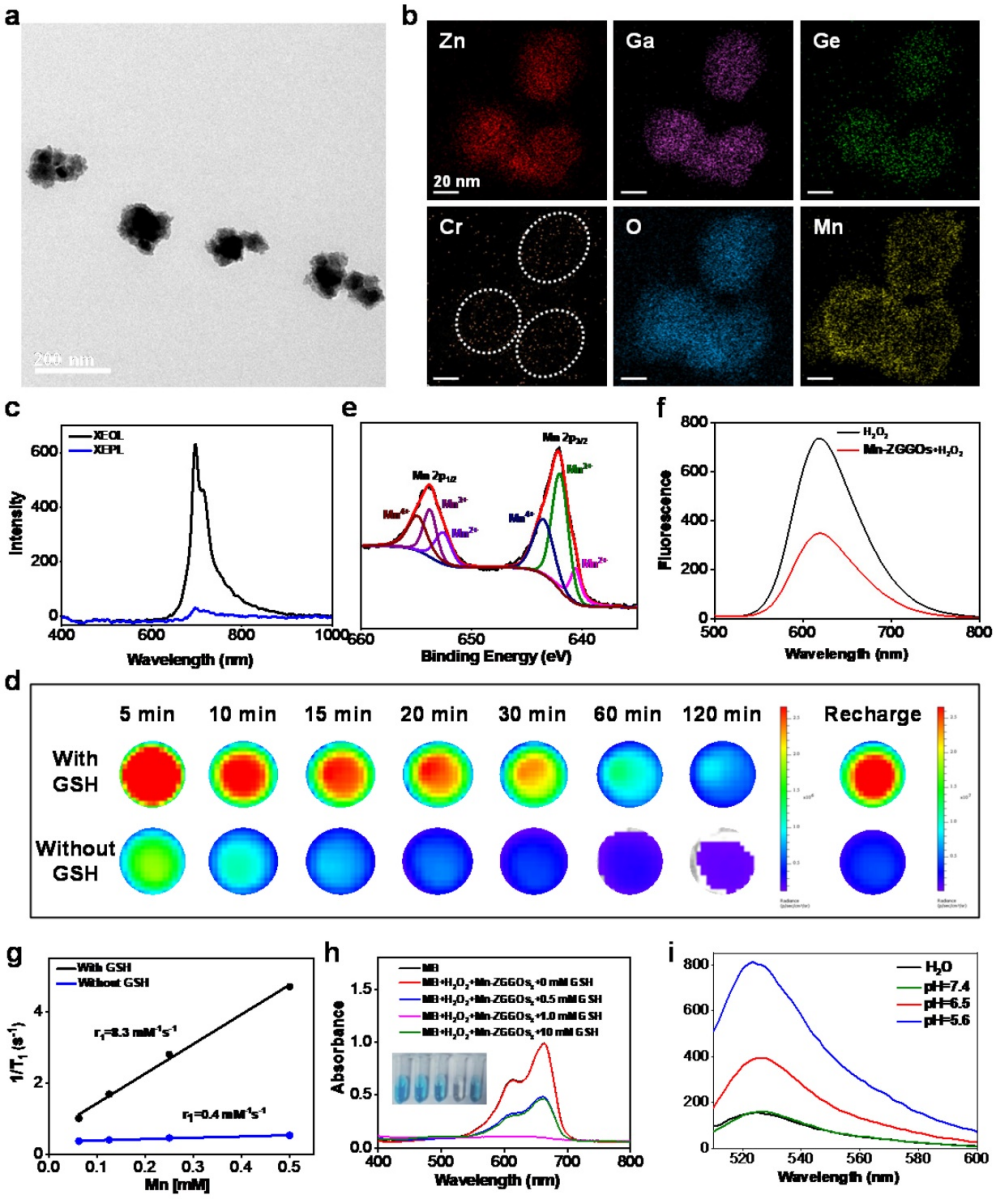
(A) TEM image of Mn-ZGGOs. (B) Elemental mapping images of Mn-ZGGOs. (C) X-ray excited optical luminescence (XEOL) and X-ray excited persistent luminescence (XEPL) spectra of Mn-ZGGOs. (D) XEPL decay images of the Mn-ZGGOs with GSH or without GSH. (E) XPS of Mn 2p for Mn-ZGGOs. (f) Fluorescence spectra of [Ru(dpp)_3_]Cl_2_ of Mn-ZGGOs with or without H_2_O_2_. (G) The r_1_ value of Mn-ZGGOs with or without 1 mM GSH in 9.4 T MR instrument. (H) UV/Vis absorption spectra and photo (inset) of MB after degradation by H_2_O_2_ plus GSH-treated Mn-ZGGOs ([HCO_3_^-^] = 25 mM, [Mn] = 0.5 mM, [H_2_O_2_] = 10 mM). (I) Fluorescence spectra of SOSG (5 μM) incubated with Mn-ZGGOs for 20 min in H_2_O or different PBS buffer (pH = 7.4, 6.5, 5.6).

ZGGO nanoparticles (ZGGOs) were prepared following our previous synthesized route 38. The ZGGOs were then stirred in a mixture of KMnO_4_ and ethanol to coat MnO_x_ layers on the surface of ZGGOs (Mn-ZGGOs). Transmission electron microscopy (TEM) imaging indicated that uniform layers were successfully formed (Figure 1A). Elemental mapping images revealed that the presence of Zn, Ga, Ge, Cr, Mn and O in Mn-ZGGOs as well as Mn circles confirmed the successful coating (Figure 1B). The MnO_x_ layer had very minor impact on ZGGO emission, which still emitted NIR luminescence at ~ 696 nm under/after X-ray irradiation attributed to the typical ^2^E → ^4^A_2_ transition of Cr^3+^ (Figures 1C, S1) 39. GSH would also be helpful to break-up the MnO_x_ layers 40. Impressively, these XEPL signals could be repeatedly activated by irradiation of X-ray; they recovered the excellent XEPL with GSH treatment (Figures 1D, S2). In addition, to prove the deep tissue-penetration of X-ray excitation and NIR emission, a 1.0 cm pork was placed between solution (Mn-ZGGOs with GSH, Mn-ZGGOs without GSH) and X-ray source (Figure S3). XEPL imaging was performed at 0, 2, and 5 min after ceasing X-ray irradiation. Strong NIR persistent luminescence signals were observed, indicating XEPL possessed deep tissue-penetration depth.

In most previous publications, MnO_x_ nanostructures were mainly employed as carriers for cancer treatment 19, 24, 41, 42. Interesting, recent studies found that MnO_x_ containing Mn (III) would generate ROS under the intrinsic acidity within tumor 34, 37. The possible mechanism revealed that the Mn valences (especially Mn(III)) impact the catalytic activity to generate ROS 37. X-ray photoelectron spectroscopy (XPS) analysis showed the presence of Mn (II), Mn (III), and Mn (IV); Mn (III) was as high as 46.5 atom% (Figure 1E). Thus, the Mn-ZGGOs would have a high activity to generate ROS.

### Responsive O_2_ production and MR imaging

We also investigated the decomposition of Mn-ZGGOs under the enviroment in tumor regions. The color changes with Mn-ZGGOs solution and TEM images indicated the rapid breakup of MnO_x_ shell in envioronment within tumor (acidic, GSH) (Figure S4). The decomposition of MnO_x_ would generate Mn^2+^ and O_2_ 43. O_2_ was produced in acidic environment (pH=5.6), detecting by the [Ru(dpp)_3_]Cl_2_ probe 44. A significant decrease in fluorescence of [Ru(dpp)_3_]Cl_2_ was shown in acidic environment (pH=5.6) (Figure 1F**)**, indicating that O_2_ was generated efficiently under the environment. Obvious brightening signals were observed in T_1_-weighted MR images with the presence of GSH versus without GSH, indicating the decomposition and release of Mn^2+^ (Figure S5). The T_1_ relaxation rate (r_1_) of Mn-ZGGOs with the presence of GSH was 8.3 mM^-1^·s^-1^ (9.4T), which was 23.2-fold higher than that without GSH (Figure 1G).

### Responsive ROS generation

Under simulated environment in tumor regions (H_2_O_2_, GSH), the absorbance of methylene blue (MB; ·OH probe) dropped sharply in the presence of Mn-ZGGOs, indicating the production of ·OH (Figure 1H). MB degradation efficiency of Mn-ZGGOs reached 51.1% when the GSH concentration was 10 mM, which is higher than that of Mn^2+^ (34.3%, Figure S6). Moreover, the fluorescence of singlet oxygen sensor green (SOSG) at 525 nm increased sharply under simulated acidic environment when incubated with Mn-ZGGOs, indicating generation of singlet oxygen (Figure 1I). These results were consistent with previous reports, and implied that the high-content of Mn(III) in Mn-ZGGOs could realize oxygen-independent production of ·OH and ^1^O_2_ simultaneously and achieve enhanced chemodynamic therapy (CDT) 37. Moreover, ZGGOs and Mn-ZGGOs showed excellent colloidal stability in PBS, DMEM, and fetal bovine serum (FBS) for further bioapplications (Figure S7).

### *In vitro* antitumor effect and mechanism

**Figure 2:**
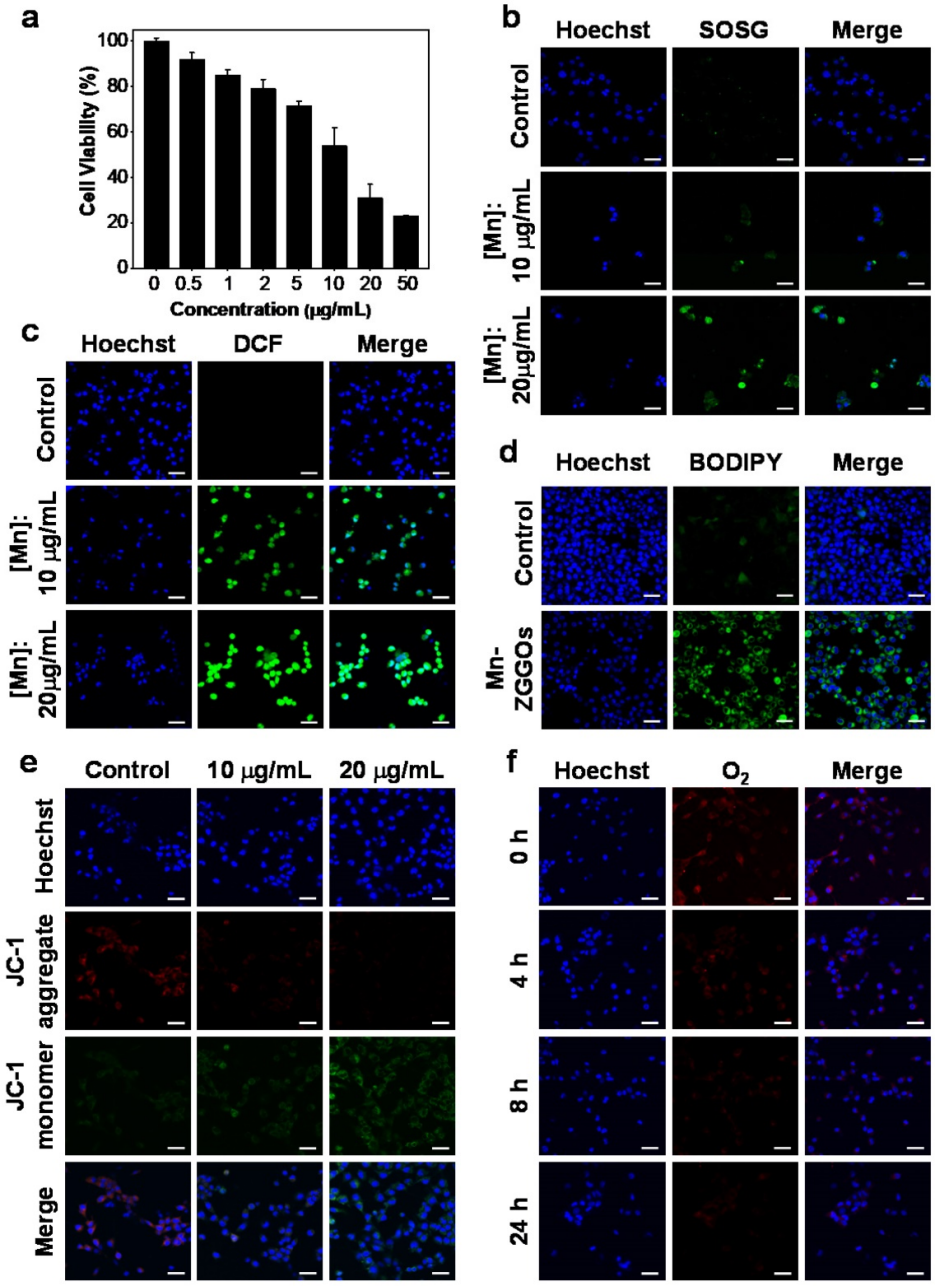
(A) Viability of U87MG cells after 24 h of incubation with Mn-ZGGOs. (B) Confocal images of U87MG cells stained by SOSG after incubating with PBS or Mn-ZGGOs for 24 h. The green fluorescence indicates the presence of ^1^O_2_. (C) Confocal images of U87MG cells stained by DCFH-DA after incubating with PBS or Mn-ZGGOs for 24 h. The green fluorescence indicates the presence of ·OH. (D) Confocal images of lipoperoxides in U87MG cells after incubation with PBS or Mn-ZGGOs for 24 h. The green fluorescence is the lipid ROS after the staining with BODIPY C11. (E) Confocal images of the changes in the mitochondrial membrane potential of U87MG cells after incubation with PBS or Mn-ZGGOs for 24 h. The red fluorescence indicates that the membrane potential is positive, and the green fluorescence indicates that the membrane potential decreases. (F) Confocal images of U87MG cells with production of O_2_ stained by [Ru(dpp)_3_]Cl_2_ after incubating with Mn-ZGGOs versus times. The blue fluorescence from Hochest 33342 indicatesthe cell nuclei in (b-e). Scale bar: 40 μm.

Encouraged by the efficient generation of ROS, we investigated the cellular ROS generation and antitumor effect *in vitro*. The U87MG cells were incubated with ZGGOs and Mn-ZGGOs, and cell viabilities revealed that the ZGGOs core had no obvious cytotoxicity. The Mn-ZGGOs at low concentrations exhibited excellent cell-killing effect with IC_50_ of 11.7 μg Mn/mL (Figures 2A, S8). Importantly, Mn-ZGGOs showed low toxicity to normal cells (L02 cells) at the high concentration ([Mn] = 0-50 μg/mL) suggesting less vulnerability to oxidative stress (Figure S9) 45.

We explored the killing mechanism of Mn-ZGGOs. After incubation with Mn-ZGGOs ([Mn] = 0, 10, and 20 μg/mL) for 24 h, U87MG cells were co-stained by Hoechst 33342 (nucleus-staining) and SOSG (^1^O_2_ indicator) or DCFH-DA (·OH indicator). The U87MG cells were incubated with Mn-ZGGOs and SOSG exhibited strong green fluorescence, indicating the generation of ^1^O_2_ (Figure 2B). More obvious green fluorescence was observed in U87MG cells incubated with Mn-ZGGOs and DCFH-DA (Figure 2C). These results demonstrated efficient production of intracellular ROS including ^1^O_2_ from reaction of Mn^3+^ and O_2_ and ·OH from the Mn^2+^-mediated Fenton-like reaction (Figure 2C). More ROS generation induced enhanced cell membrane damage 46. BODIPY C11 showed significant green fluorescence implying high-levels of lipid peroxidation (Figure 2D).

The mitochondrion is vulnerable to the accumulated ROS in the tumor cell resulting in early cellular apoptosis and changes in the mitochondrial membrane potential 47. Therefore, we assessed the cell apoptosis by staining with a mitochondrial membrane potential fluorescence probe, JC-1. The red fluorescence decreased and green increased gradually, indicating increased cell damage by ROS (Figure 2E). Furthermore, the decomposition of MnO_x_ would produce O_2_ and Mn^2+^ and expose the ZGGOs. Figure 2F shows that the red fluorescence of [Ru(dpp)_3_]Cl_2_ gradually decreased as the incubation time increased, indicating continuous O_2_ supply of Mn-ZGGOs in U87MG cells, this further relieved the tumor hypoxia. *In vitro* T_1_-weighted MR imaging of U87MG cells was evaluated by incubation with different concentrations of Mn-ZGGOs. After 24 h, the U87MG cells precipitated to the bottom of the tubes. Versus the control group, significant MR signals were observed in the U87MG cells incubated with Mn-ZGGOs, which showed the concentration-dependent behavior (Figure S10). Moreover, the exposed ZGGOs could recover the excellent XEPL by decomposition of MnO_x_. Figure S11 shows that the U87MG cells were incubated with Mn-ZGGOs for 24 h and exhibited significantly more enhanced XEPL signals. These results demonstrated that Mn-ZGGOs would generate ROS (^1^O_2_, ·OH) during the decomposition process and release imaging agents (Mn^2+^, afterglow) as well as release O_2_ to decrease tumor hypoxia.

### *In vivo* biodistribution and biosafety

**Figure 3:**
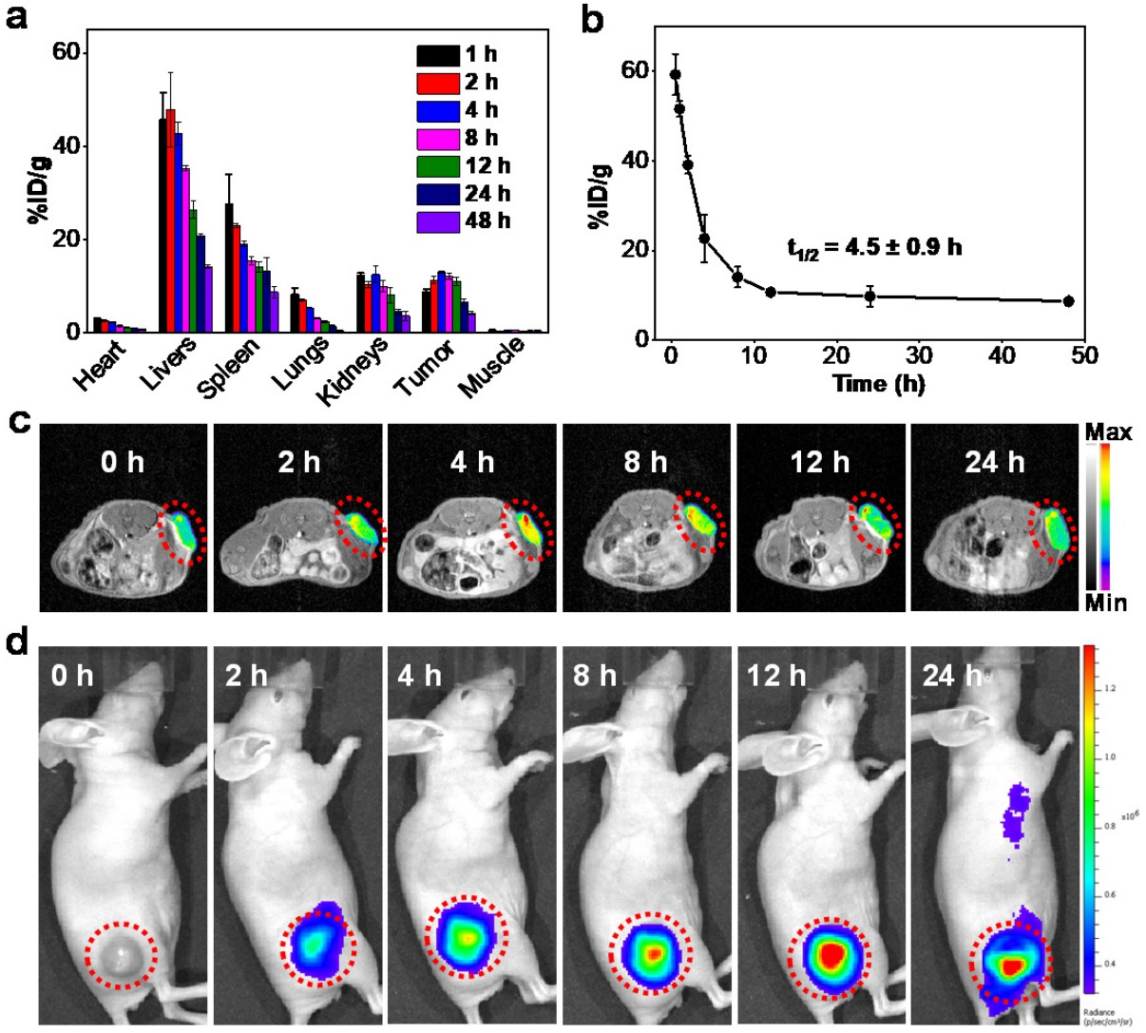
(A) Biodistribution of Mn-ZGGOs in major organs and tumors after intravenous administration at various time intervals (1, 2, 4, 8, 12, 24, and 48 h). The Mn-ZGGOs concentrations were normalized as the percentage of the injected dose of Mn element per gram of each organ (%ID g^-1^). (B) Time course of blood levels of Mn-ZGGOs levels following intravenous injection. The half-life time (t_1/2_) was calculated to be 4.5 ± 0.9 h. (C) *In vivo* T_1_-weighted MR images of mice injected intravenously with Mn-ZGGOs. (D) *In vivo* XEPL imaging of tumor-bearing mice after intravenous injection of Mn-ZGGOs.

The *in vivo* blood-clearance behaviors and biodistribution were assessed by measuring the concentrations of Mn in dissolved tissues using inductively coupled plasma mass spectrometry (ICP-MS). The accumulation of Mn-ZGGOs in tumor tissue was as high as 13.2 % of the injected dose of Mn element per gram tissue (%ID g^-1^) at 4 h post-injection (Figure 3A). The pharmacokinetics showed that the half-life of Mn-ZGGOs was 4.5 ± 0.9 h (Figure 3B), which would benefit effective accumulation of Mn-ZGGOs into tumors. Furthermore, the long-term biocompatibility was evaluated by intravenous injection of Mn-ZGGOs ([Mn] = 1 mg/kg) into healthy mice. Blood biochemistry and hematology analysis showed no significant difference in hepatic function, kidney function, and blood indexes before and after injection of Mn-ZGGOs, indicating that Mn-ZGGOs had no systematic toxicity (Figures S12, S13).

### *In vivo* imaging of U87MG tumor

The decomposition of MnO_x_ *in vivo* was first evaluated by injecting Mn-ZGGOs intratumorally in U87MG tumor-bearing mice as well as normal subcutaneous tissue (Figure S14, black circle: tumor, red circle: normal subcutaneous tissue, Figure S15). In tumors, obvious T_1_-MR signals and US signals were observed confirming the release of Mn^2+^ for MR imaging and generation of O_2_ for US imaging.

Due to the effective response of the environment in tumor regions, we further studied the tumor-diagnosis of Mn-ZGGOs via *in vivo* imaging. The Mn-ZGGOs were intravenously injected in U87MG tumor-bearing mice ([Mn] = 1 mg/kg): Obvious T_1_-MR signals (red and yellow signals in tumors) were observed and reached a peak at 4 h post-injection, suggesting effective accumulation and respond decomposition of Mn-ZGGOs in U87MG tumors (Figures 3C, S15A); these findings were confirmed with biodistribution analysis.

As the ZGGOs core of Mn-ZGGOs also had persistent luminescence property, we then irradiated the tumor regions using X-ray to study the *in vivo* XEPL imaging after 0, 2, 4, 8, 12, and 24 h post-injection. After X-ray excitation (0.1 Gy), the X-ray excited persistent luminescence (XEPL) gradually increased over time and reached a peak at 12 h post-injection (Figures 3D, S17B). The difference was due to the restoration of persistent luminescence via break-up of the MnO_x_ coating in acidic environment in tumor regions.

### *In vivo* U87MG tumor therapy

**Figure 4:**
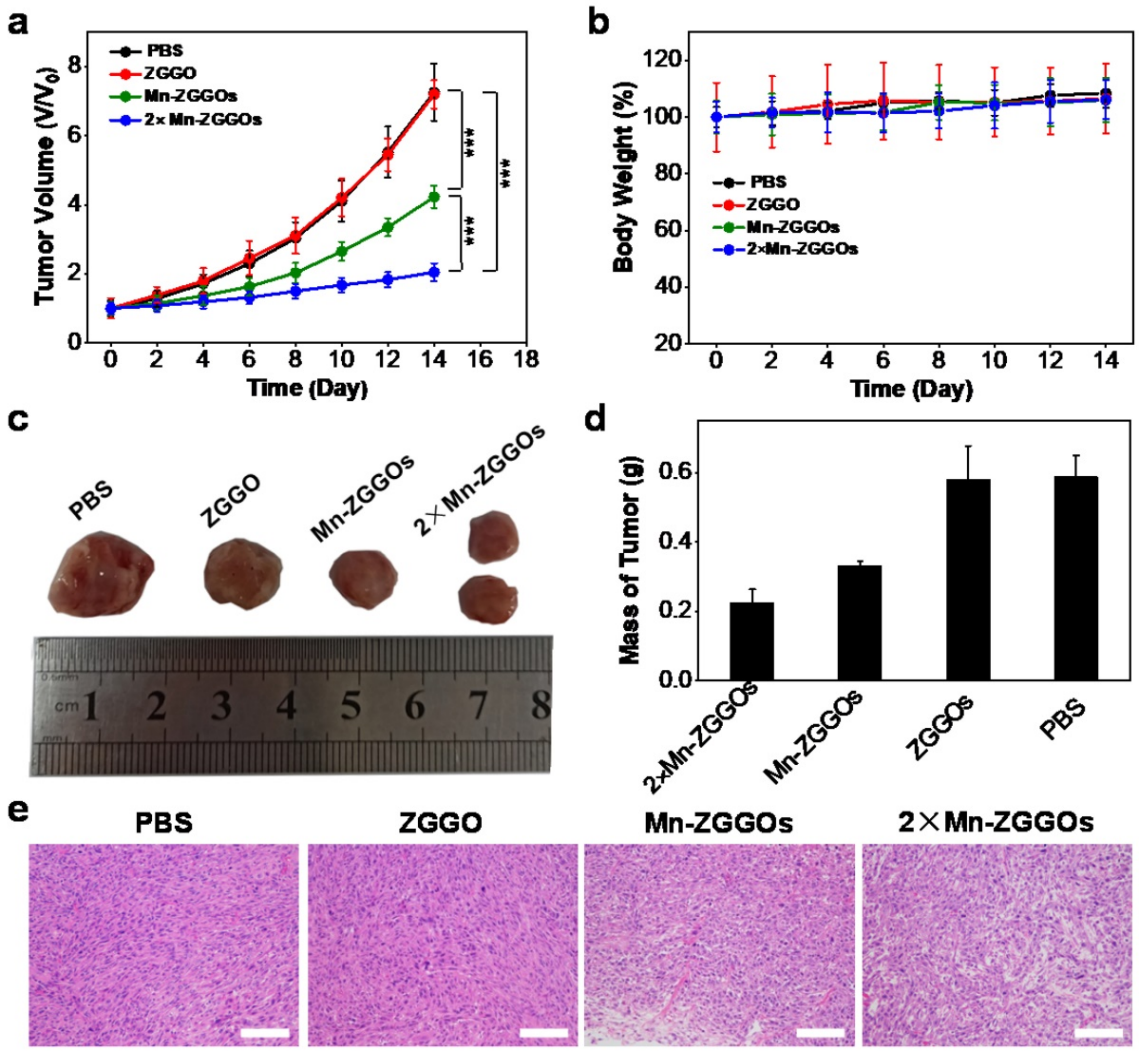
(A) Tumor volume curves and (B) Body weight growth curves of four groups of U87MG tumor-bearing mice at 14 days after intravenous injection with different formulations. Error bars are based on mean ± standard deviation (n = 5), ***P<0.001. (C) Typical photographs of excised tumors at day 14 after different treatments. (D) Relative tumor mass after different treatments on day 14. (E) H&E staining of tumor tissues after different treatments on day 14. Scale bar: 50 μm.

Following the *in vivo* study of distribution and decomposition of Mn-ZGGOs, the *in vivo* anticancer efficacies on U87MG tumor-bearing mice were evaluated. When the tumor volumes reached to 60 mm^3^, the mice were randomly allocated into four groups (n=5 per group): (1) PBS, (2) ZGGO (6.3 mg/kg), (3) Mn-ZGGOs (8.9 mg/kg, [Mn] = 1 mg/kg), and (4) 2×Mn-ZGGOs (17.8 mg/kg, [Mn] = 2 mg/kg). The tumor size and body weight were monitored every two days after injection. Versus controls, the Mn-ZGGOs groups inhibited tumor growth with the inhibition rate of 41.8 % at an Mn dose of 1 mg/kg (Figure 4A). A significantly enhanced tumor inhibition effect (inhibition rate: 71.8 %) was achieved in the 2-fold Mn-ZGGOs. The typical photographs of tumor tissue and the tumor masses at the end of treatment showed a consistent suppressive trend with tumor growth (Figure 4C, 4D). In addition, hematoxylin and eosin (H&E) staining analysis revealed that the tumor suffered more serious damage in CDT groups than those in PBS and ZGGO groups (Figure 4E). Both body weight and H&E staining of the major organs, including hearts, livers, spleens, lungs and kidneys, had no significant change/damage, indicating that Mn-ZGGOs had minor systemic toxicity (Figures 4B, S18). These results demonstrated that the procedure would produce Mn(III)-rich Mn-ZGGOs, and generate abundant ROS for satisfactory enviroment-activated chemodynamic therapeutics and imaging agents to with value in guilding precision cancer therapy.

## Conclusion

In summary, we report an easy and novel method to prepare Mn(III)-rich nanotheranostics of Mn-ZGGOs. Under the environment in tumor regions (acidic pH, high H_2_O_2_, and GSH levels), Mn-ZGGOs could react with the GSH/H_2_O_2_ to release the agents for imaging and treatment. The released Mn(III)-pool could directly react with O_2_ to generate ^1^O_2_; Mn(II) reacted with H_2_O_2_ to generate ·OH. The released *in situ* Mn(II), O_2_ and ZGGOs can be employed as MR, US and persistent luminescence diagnostics to guide precision cancer therapy. The tumor cell-killing mechanism *in vitro* and *in vivo* was confirmed systematically by detection of ROS, lipid peroxidation and mitochondrial membrane potential changes and tumor inhibition. Overall, this Mn^3+^-rich Mn-ZGGOs nanotheranostics design promotes the ultrasensitive, X-ray reactivated and high spatial resolution imaging. There is light-free generation of singlet oxygen and hydroxyl radicals for enhanced tumor chemodynamic therapy.

## Supplementary Material

Supplementary materials and figures.

## Abbreviations

XEPL: X-ray excited persistent luminescence
MnO_x_: manganese oxide
ZGGO: chromium-doped zinc gallogermanate
Mn-ZGGOs: manganese oxide-coated chromium-doped zinc gallogermanate nanoparticles
MR: magnetic resonance
ROS: reactive oxygen
O_2_: oxygen
^1^O_2_: singlet oxygen
·OH: hydroxyl radical
NIR: near-infrared
GSH: glutathione
CDT: chemodynamic therapy
TEM: transmission electron microscopy
XPS: X-ray photoelectron spectroscopy
MB: methylene blue
SOSG: singlet oxygen sensor green
FBS: fetal bovine serum
ICP-MS: inductively coupled plasma mass spectrometry
H&E: hematoxylin and eosin
