# Supplementary material for: Mn3+-rich oxide/persistent luminescence nanoparticles achieve light-free generation of singlet oxygen and hydroxyl radicals for responsive imaging and tumor treatment
Source: Theranostics. 2021 May 25;11(15):7439–49. doi: 10.7150/thno.62437 (PMC8210605; doi:10.7150/thno.62437)
Supplement: Supplementary file 1 — Supplementary materials and figures. [file thnov11p7439s1.pdf]

Supporting Information for

Mn^{3+}-rich oxide/persistent luminescence nanoparticles achieve light-free generation of singlet oxygen and hydroxyl radicals for responsive imaging and tumor treatment

Dandan Ding[1], Yushuo Feng[1], Ruixue Qin[1], Shi Li[1], Lei Chen[1], Jinpeng Jing[1], Chutong Zhang[1], Wenjing Sun[1], Yimin Li[2,*], Xiaoyuan Chen[3,*], Hongmin Chen [1,*]

1. State Key Laboratory of Molecular Vaccinology and Molecular Diagnostics & Center for Molecular Imaging and Translational Medicine, School of Public Health, Xiamen University, Xiamen 361102, China

E-mail: hchen@xmu.edu.cn

2. Department of Radiation Oncology, Cancer Center, the First Affiliated Hospital of Xiamen University, Xiamen 361003, China

E-mail: lym05@xmu.edu.cn

3. Departments of Diagnostic Radiology and Surgery, Clinical Imaging Research Centre, Centre for Translational Medicine, Nanomedicine Translational Research Program, NUS Center for Nanomedicine, Yong Loo Lin School of Medicine, Departments of Chemical and Biomolecular Engineering, and Biomedical Engineering, Faculty of Engineering, National University of Singapore, Singapore

E-mail: chen.shawn@nus.edu.sg

Materials: Zinc nitrate hexahydrate ($Zn(NO_3)_2·6H_2O$, 99%), chromium nitrate nonahydrate ($Cr(NO_3)_3·9H_2O$, 99.99%), 4-nitrophenyl chloroformate 3-(4,5-dimethylthiazol-2-yl)-2,5-diphenyltetrazolium bromide (MTT), singlet oxygen sensor green (SOSG) and hydroxyphenyl fluorescein (DCFH-DA) were purchased from Sigma-Aldrich (MO, USA). Gallium nitrate hydrate ($Ga(NO_3)_3·xH_2O$, 99.9%), germanium dioxide (GeO_2, 99%), hydrogen peroxide (H_2O_2, 30 wt.%), sodium hydroxide (NaOH) and 9,10-anthracenediylbis (methylene) dimalonic acid (ABDA, 99%) were purchased from Aladdin. Ammonia aqueous solution (28 wt.%) and ethanol were supplied by Sinopharm Chemical Reagent Co., Ltd. Methylene Blue was purchased from ACROS. Ultrapure water was obtained from a Millipore autopure system. Potassium permanganate was purchased from Alfa. Hoechst 33342 and BODIPY 581/591 C11 were purchased from Thermo Fisher Scientific (Waltham, MA). JC-1 was purchased from MedChemExpress (MCE). All chemicals were used without further purification unless noted. U87MG cells were obtained from Institute of Biochemistry and Cell Biology (Shanghai, China).

Characterization: Crystal phase composition was determined by X-ray diffraction (XRD) measurement performed on an X-ray powder diffractometer (Bruker, D8 ADVANCE) with Cu Kα radiation (λ = 1.5406 Å) at 40 kV and 40 mA. Transmission electron microscopy (TEM) images were obtained using a microscope at an acceleration voltage of 200 kV (FEI Tecnai G2 F30). Persistent luminescence imaging was conducted using an IVIS in vivo imaging system (Caliper IVIS, Lumina II). X-ray excited persistent luminescence (XEPL) was measured using an Ocean Optics QE Pro optical bench. The mini-X X-ray tube (Amptek Inc.) was set at 50 kV and 70 μA. The fluorescence images of cells were taken on a laser scanning confocal microscopy (Olympus FV1200, Japan). The magnetic resonance imaging (MRI) was performed on a 9.4T BioSpec MRI (Bruker, Germany).

Synthesis of ZGGO: ZGGO:Cr were prepared by hydrothermal reaction and calcinating treatment. Firstly, 1.2 mmol $Zn(NO_3)_2$, 1.6 mmol $Ga(NO_3)_3$, 0.2 mmol Na_2GeO_3 and 0.005

mmol $Cr(NO_3)_3$ were dissolved in 25 mL of deionized water. Ammonium hydroxide (28 wt %) was added to adjust the pH value to ~ 8. Then, the mixture was vigorously stirred for 2 h at room temperature (r.t.). After that, the milky suspension solution was transferred to a 50 mL Teflon-lined autoclave and reacted at 200 °C for 12 h. The precipitates were collected by centrifugation and washed for three times with deionized water. Finally, the precipitates were dried in vacuum at 60 °C and annealed at 850 °C in air for 4 h to obtain persistent luminescence property. The obtained powder was wet ground in 5 mM NaOH solution, and collected by centrifugation and washed for three times with deionized water.

·OH generation by Mn^{2+}-mediated Fenton-like reaction: 25 mM $NaHCO_3$ solution containing 10 μg/mL MB, 10 mM H_2O_2, and 0.5 mM $MnCl_2$ was allowed to mix at 37 °C for 30 min. The ·OH-induced MB degradation was monitored by the absorbance change at 665 nm.

Cell culture: U87MG cells were cultured in DMEM medium containing 10% fetal bovine serum (Gibco), penicillin (100 U/ml), and streptomycin (100 μg/ml) in a humidified atmosphere with 5% CO_2 at 37 °C.

In vitro T_1-MR imaging: U87MG cells (1×10^6 cells per well) were seeded into 6-well plates and allowed to grow overnight. The fresh medium containing Mn-ZGGOs ([Mn] = 5, 10 μg/mL) was added. After incubation for 24 h, the U87MG cells were washed with PBS for three times and were precipitated at the bottom of tube after centrifugation. The MR imaging was performed on 9.4 T clinical MRI instrument.

In vitro XEPL imaging: U87MG cells (1×10^4 cells per well) were seeded into 96-well plates and allowed to grow overnight. The U87MG cells were incubated with Mn-ZGGOs ([Mn] = 20 μg/mL) for 0 h and 24 h. The XEPL imaging was performed on an IVIS Lumina II imaging system.

Animal experiments: Female Balb/c nude mice (18~22 g) were purchased from Shanghai SLAC Laboratory Animal Co. Ltd (Shanghai, China). All of the *in vivo* experiments were

carried out according to the criteria of the National Regulation of China for Care and Use of Laboratory Animals. Balb/c nude mice were subcutaneously injected with U87MG cells (5×10^6 cells/mouse) in the right flank to establish the U87MG tumor-bearing mice model.

Serum chemistry and hematology cell analysis: Balb/c mice were intravenously injected with Mn-ZGGOs ([Mn] = 1 mg/kg). Blood samples were collected for serum chemistry and blood cell analysis at day 0, day 3, and day 7 post-injection. Alkaline phosphatase (ALP), albumin (ALB), cholinesterase (CHE), and creatinine (CREA-S) in serum were measured using an Automatic Biochemistry Analyzer (Mindray, BS-220). White blood cell (WBC), hemoglobin (HGB), red blood cell (RBC), hematocrit (HCT), mean corpuscular volume (MCV), mean corpuscular hemoglobin (MCH), mean corpuscular hemoglobin concentration (MCHC), RBC distribution width (RDW-CV and RDW-SD), platelets (PLT), mean platelet volume (MPV), platelet distribution width (PDW) were measured using an Automatic Tri-classification Blood Cell Analyzer (Mindray, BC-2600).

Responsive MR and US imaging: To study the decomposition of MnO_x shell and production of O_2 in the environment within tumor, Mn-ZGGOs NPs were injected intratumorally into U87MG tumor-bearing mice. MR and US imaging was performed at different time points after injection.

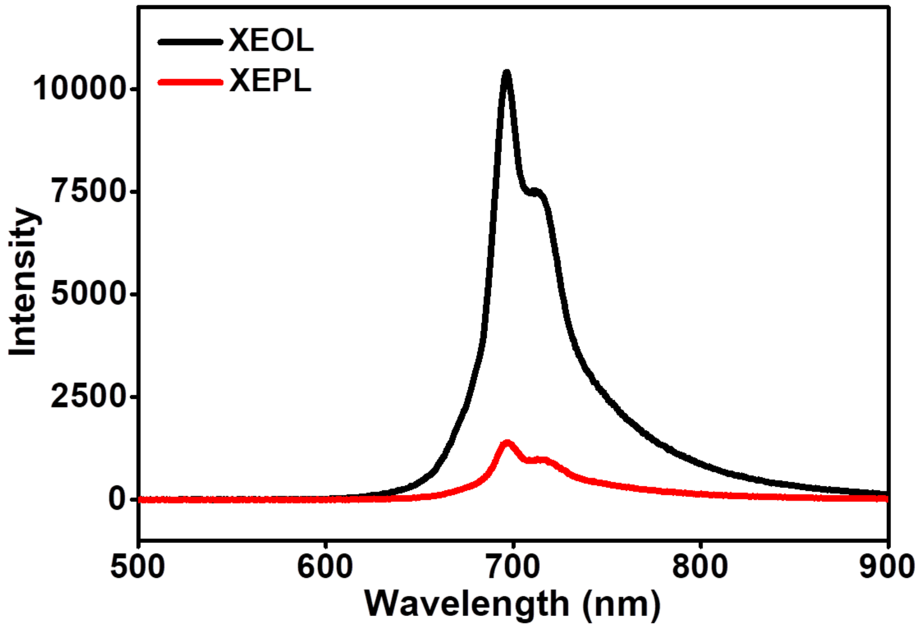

Figure S1. XEOL and XEPL spectra of ZGGO nanoparticles.

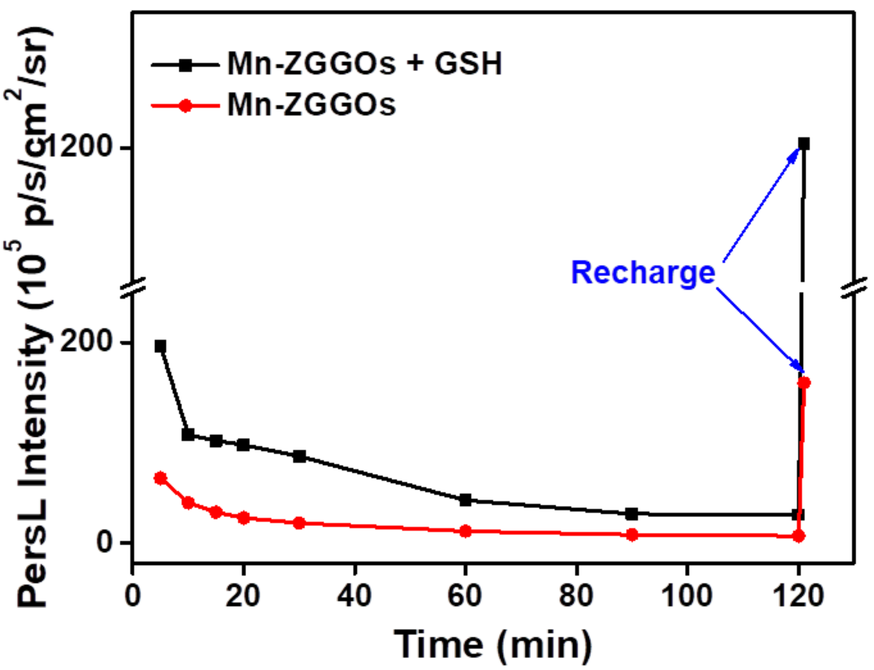

Figure S2. XEPL intensity of Mn-ZGGOs with GSH or without GSH.

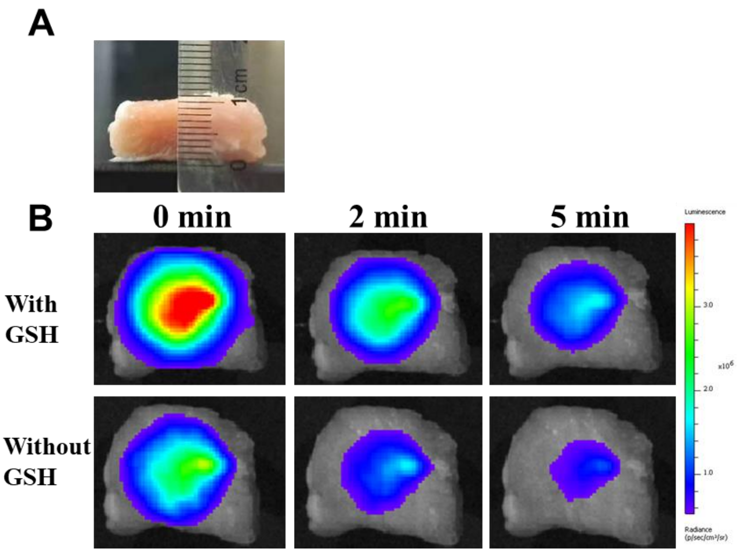

Figure S3. (A) The photo of 1.0 cm pork. (B) X-ray excited NIR persistent luminescence was detected under 1.0 cm thick pork. The images were acquired immediately and after 2 min and 5 min X-ray irradiation. The pork slice remained on top of the nanoparticles throughout the experiment.

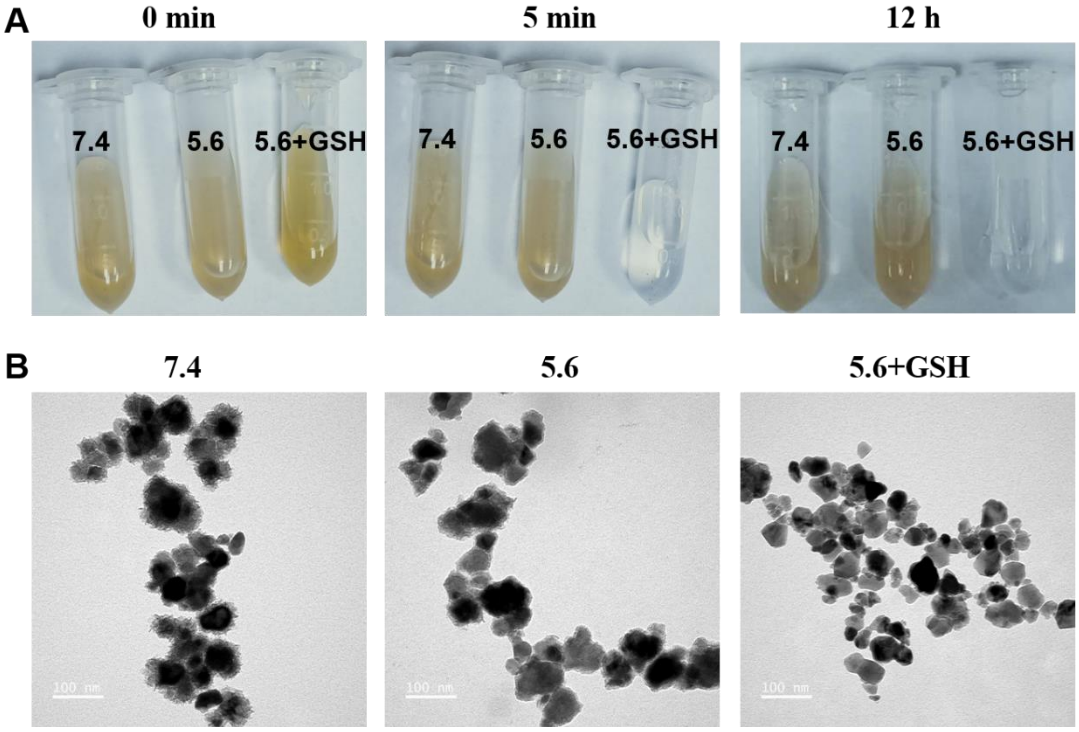

Figure S4. (A) Photos of Mn-ZGGOs after incubation in different buffers with pH 7.4, pH 5.6 and pH 5.6 plus GSH. (B)TEM images of Mn-ZGGOs after incubation for 12 h in different buffers with pH 7.4, pH 5.6 and pH 5.6 plus GSH.

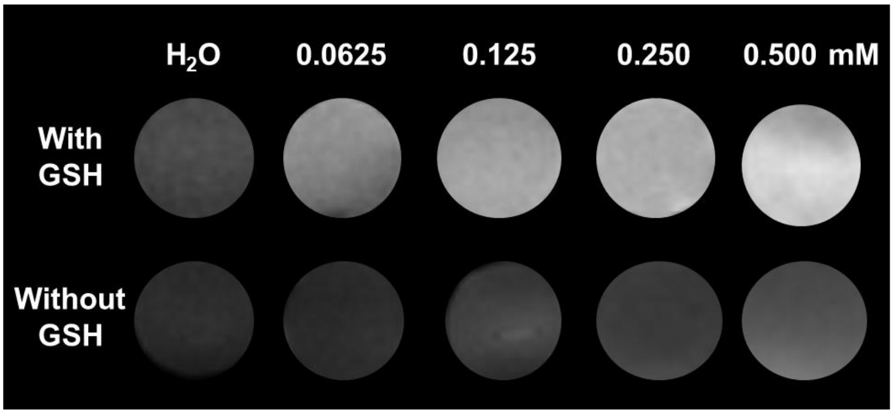

Figure S5. Phantom study of Mn-ZGGOs at 9.4 T.

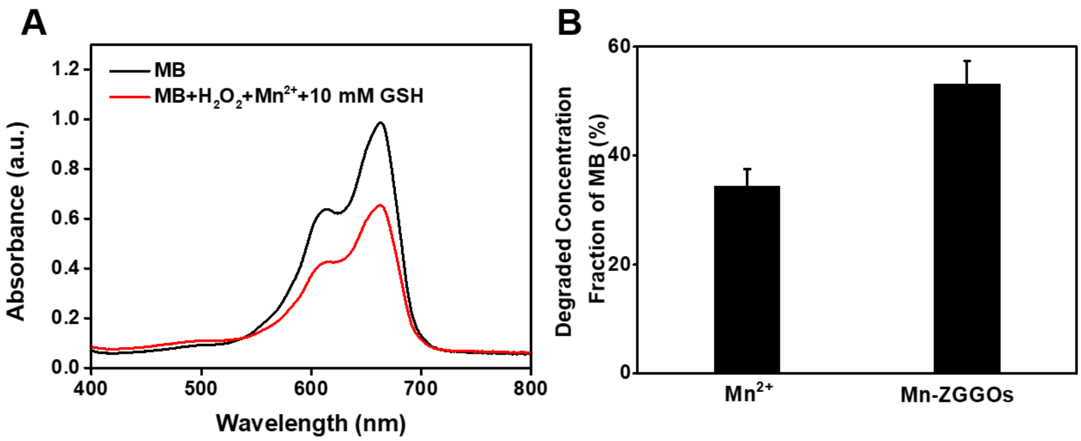

Figure S6. (A) UV/Vis absorption spectra of MB after degradation by the Mn^{2+}-mediated Fenton-like reaction in the presence of GSH ($[HCO_3^-]$ = 25 mM, [Mn] = 0.5 mM, $[H_2O_2]$ = 10 mM). (B) Bar graph showing the degradation percent of MB by H_2O_2 plus Mn^{2+} or Mn-ZGGOs in the presence of 10 mM GSH.

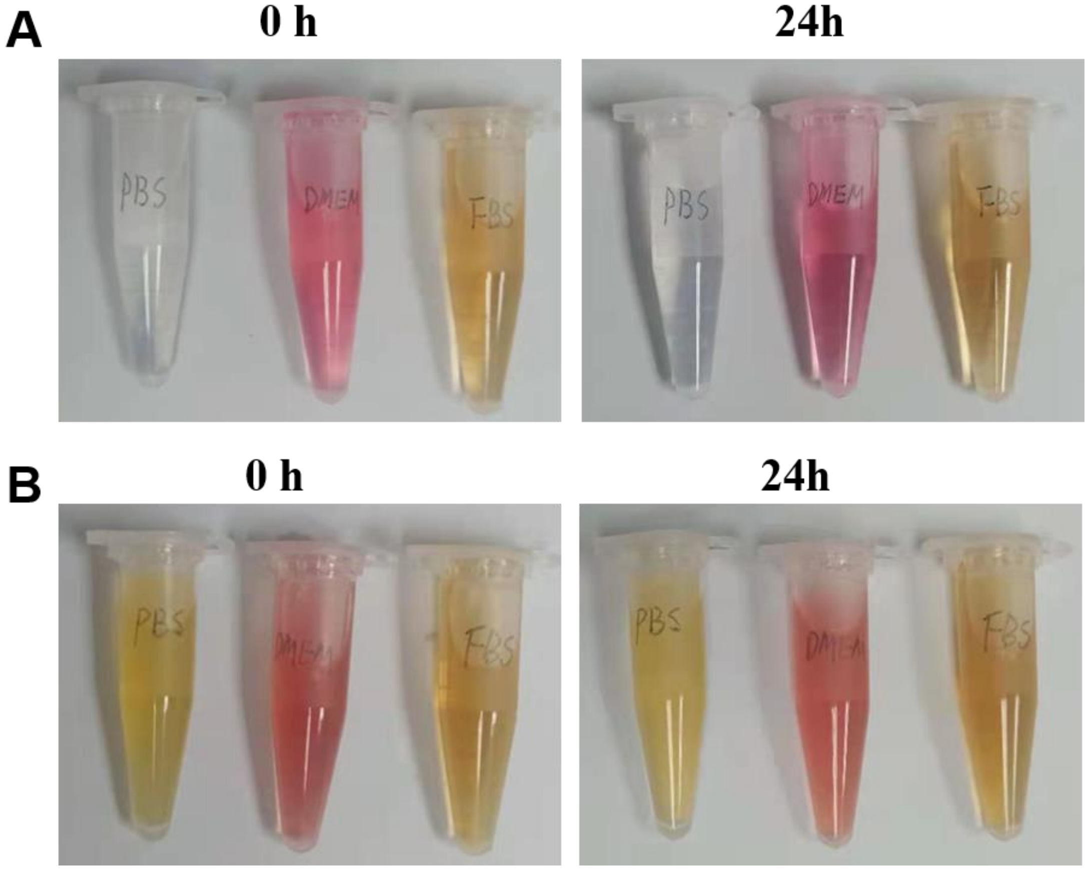

Figure S7. (A) Stability of ZGGOs dispersed in PBS, DMEM and FBS. (B) Stability of Mn-ZGGOs dispersed in PBS, DMEM and FBS.

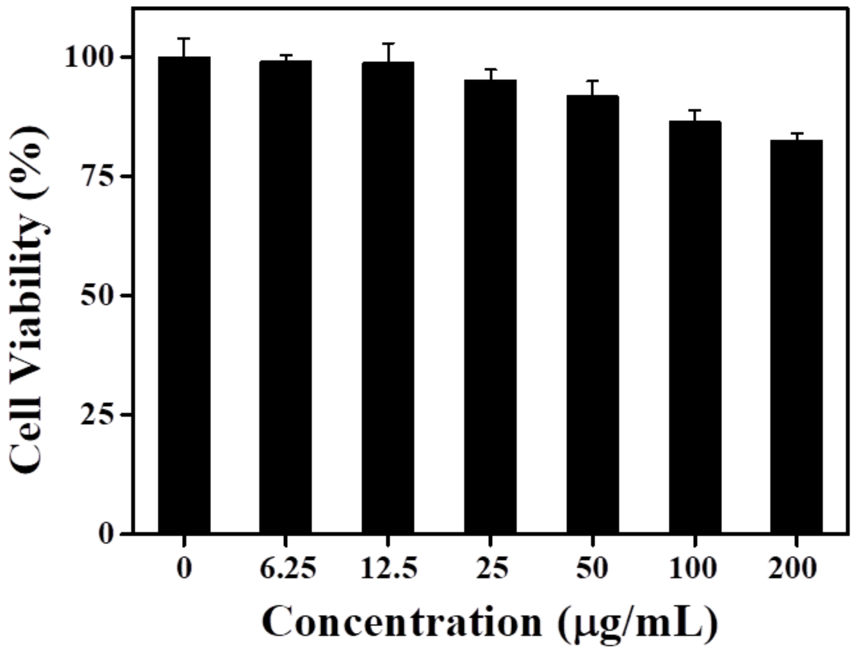

Figure S8. Viability of U87MG cells after 24 h of incubation with ZGGO nanoparticles.

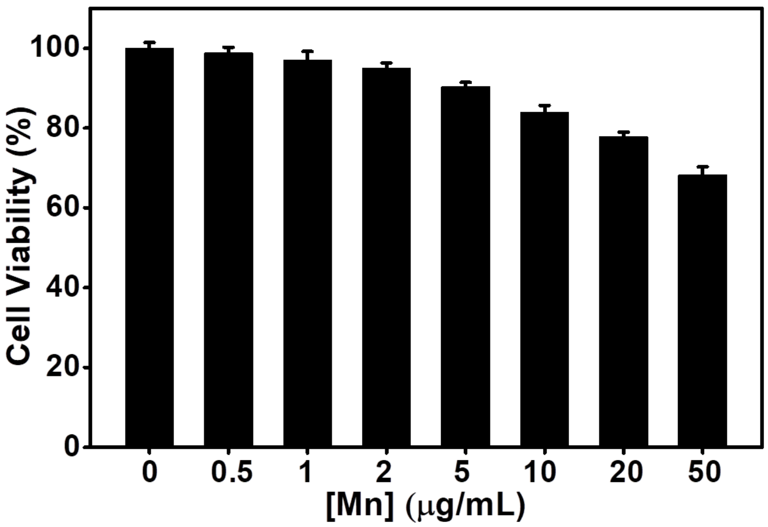

Figure S9. Viability of L02 cells after 24 h of incubation with Mn-ZGGOs.

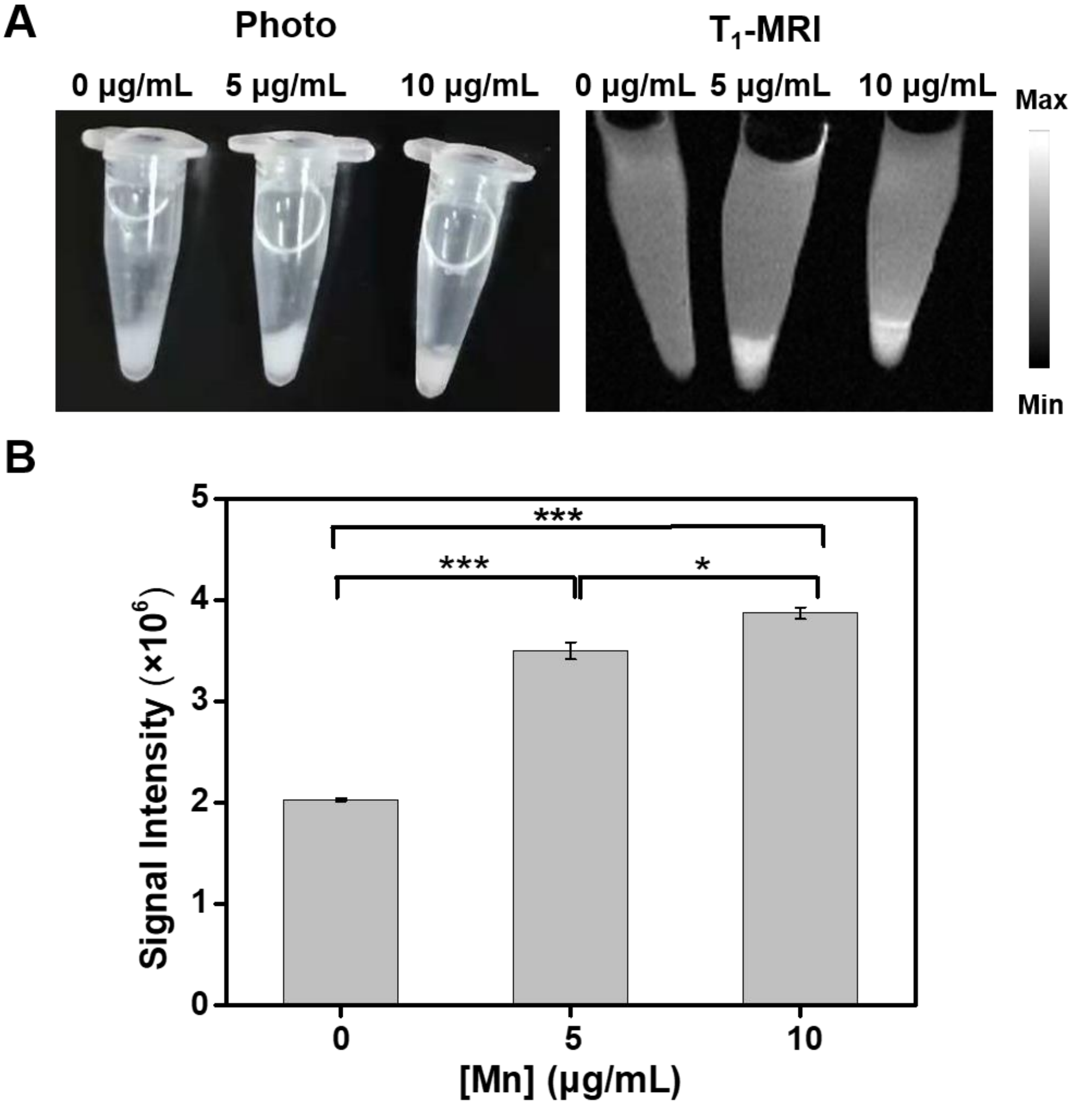

Figure S10. (A) T_1-weighted MR imaging of U87MG cells after incubation with Mn-ZGGOs for 24 h. (B) T_1-MR imaging signal intensity of U87MG cells after incubation with Mn-ZGGOs for 24 h.

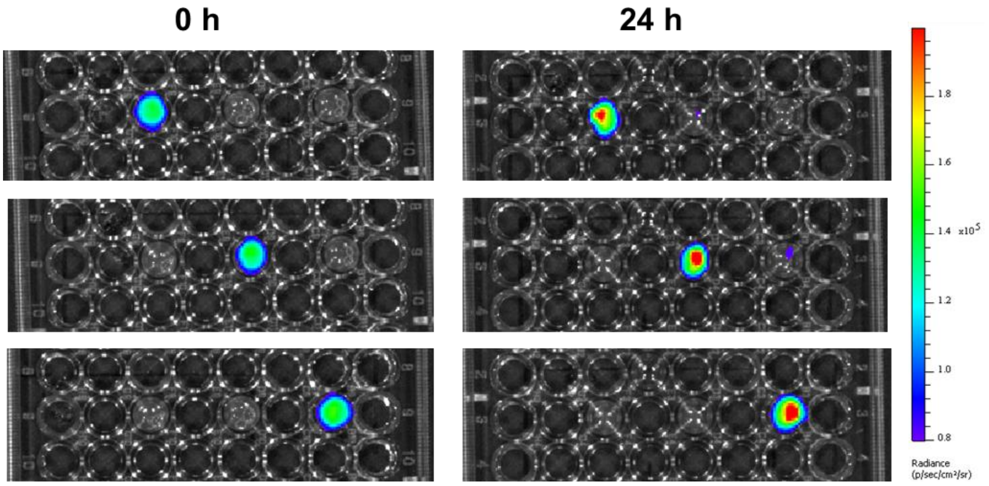

Figure S11 XEPL imaging of U87MG cells after incubation with Mn-ZGGOs for 0 h and 24 h.

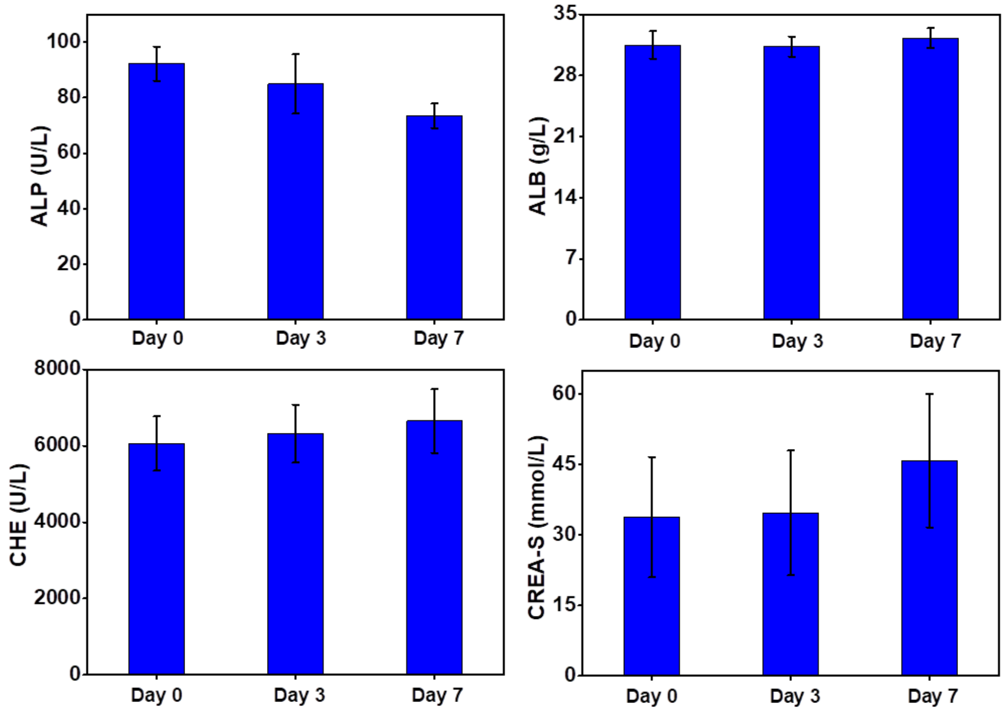

Figure S12. Serum chemistry of mice treated with Mn-ZGGOs. ALP, alkaline phosphatase; ALB, albumin; CHE, cholinesterase; CREA-S, creatinine. Blood samples were collected for serum chemistry analysis on day 0, day 3 and day 7 after treatment.

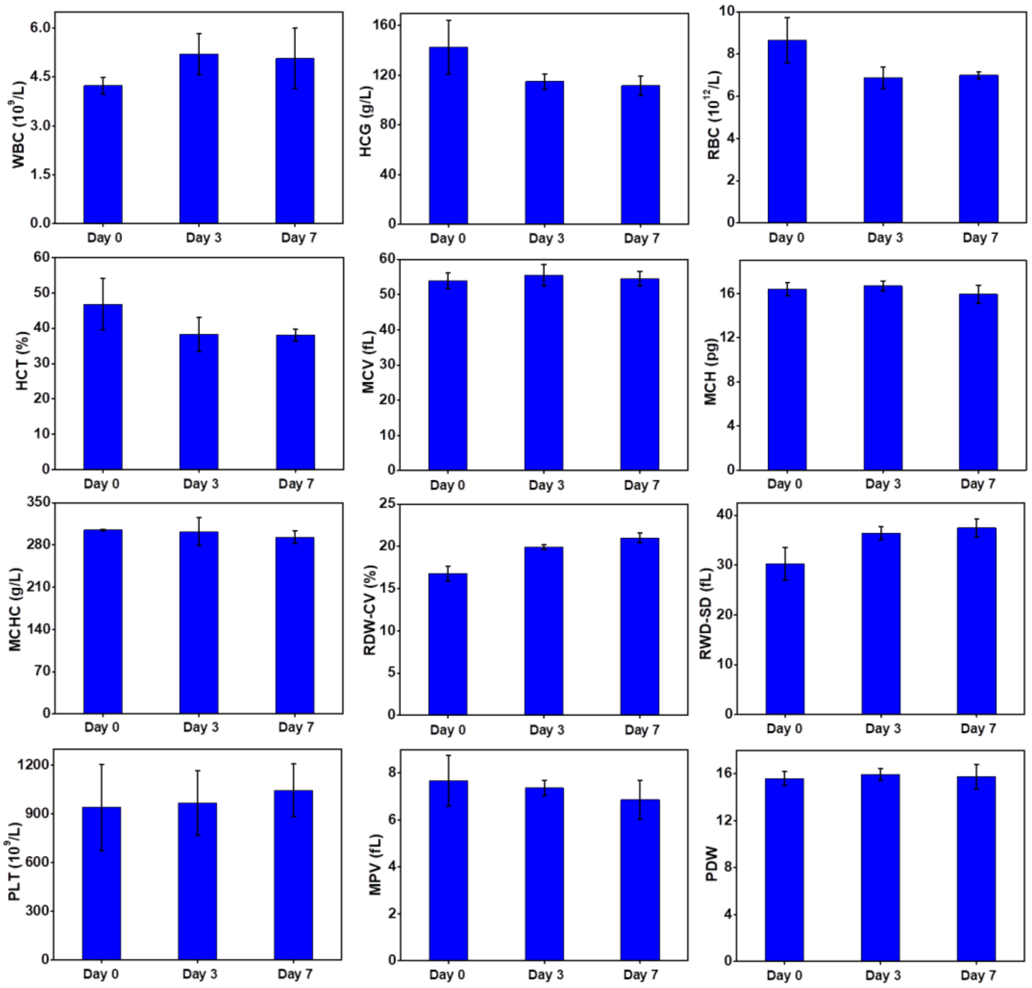

Figure S13. Hematological analysis of mice treated with Mn-ZGGOs. WBC, white blood cell; HGB, hemoglobin; RBC, red blood cell; MCV, mean corpuscular volume; MCH, mean corpuscular hemoglobin; MCHC, mean corpuscular hemoglobin concentration; RDW-SD, standard deviation of RBC distribution width; HCT, hematocrit; PLT, platelets; MPV, mean platelet volume. Blood samples were collected for hematological analysis on day 0, day 3 and day 7 after treatment.

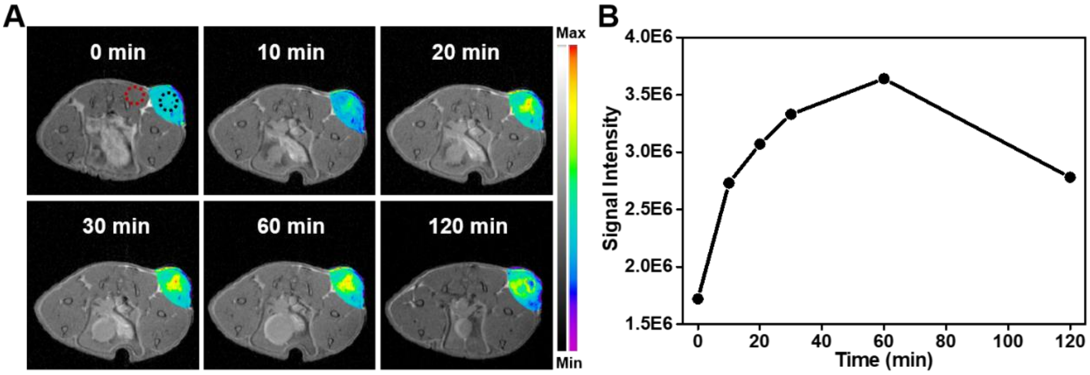

Figure S14. (A) T_1-weighted images of normal and tumor subcutaneous tissues before and after injection with Mn-ZGGOs at different time points. (B) T_1-MRI signal intensity before and after injection of Mn-ZGGOs.

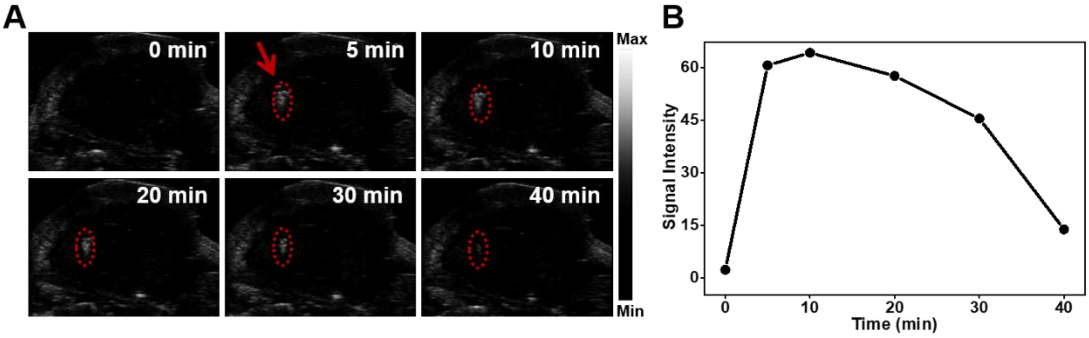

Figure S15. (A) Ultrasound images showing the generation of O_2 by intratumor injection of Mn-ZGGOs at different time points. (B) Relative ultrasound signal intensity before and after injection of Mn-ZGGOs.

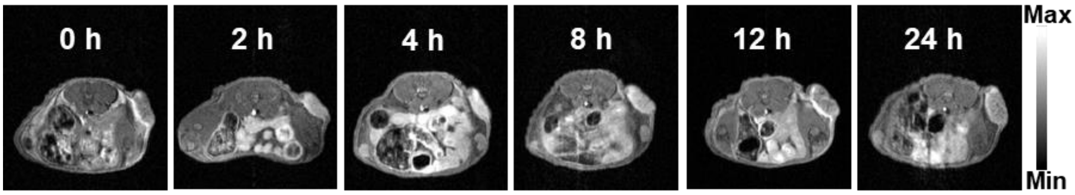

Figure S16. In vivo T_1-weighted MR images of mice injected intravenously with Mn-ZGGOs.

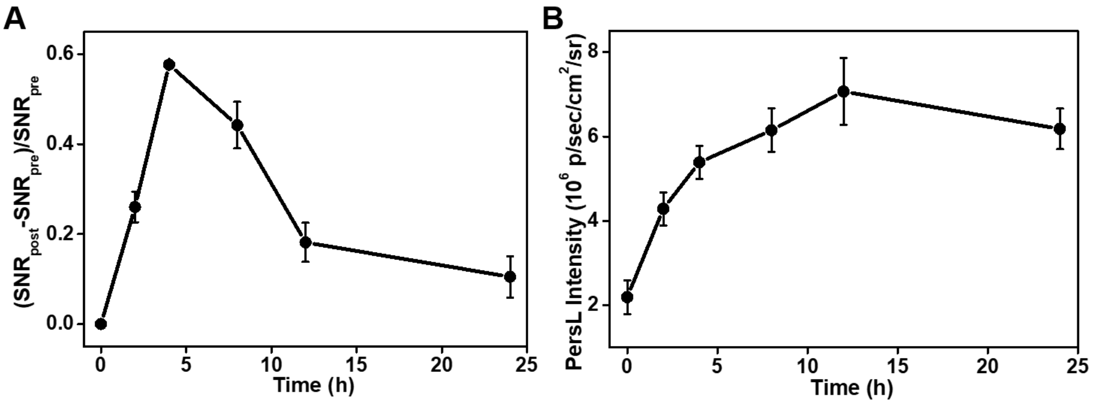

Figure S17. T_1-MR imaging signals (A) and fluorescence signals (B) in tumors at 0, 2, 4, 8, 12 and 24 h post-injection intravenously of Mn-ZGGOs in U87MG tumor-bearing mice.

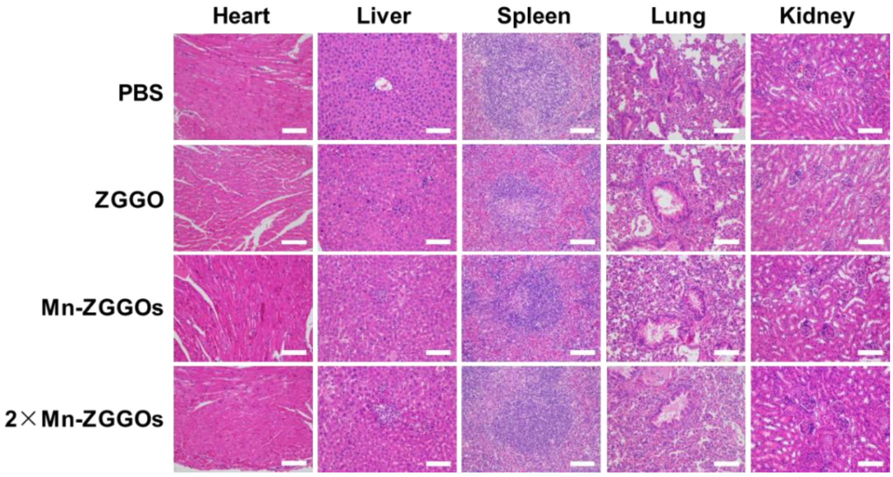

Figure S18. Hematoxylin & eosin (H&E)-stained slice images of major organs collected from different groups after 14 days of treatment. (Scale bar: 100 μm)
